# Monthly Summary

**Published:** 1870-02

**Authors:** 


### MONTHLY SUMMARY.

*Death from Inhalation of Bi-Chloride of Methylene.*—An actor of some repute, Mr. Chas. Verner. aged 39 years, admitted into Charing-Cross Hospital with a malignant disease of the upper jaw, from which there seemed but faint hope of recovery, without or with operative interference, decided to take the chances of an operation, and on October 15, 1869, after due preparation, the bi-chloride of methylene was administered with care and skill. Within two minutes of its administration and before the operation was commenced, the patient fell back and died. The galvanic apparatus and other appliances were resorted to, but life was extinct. An inquiry seemed to indicate the result as one of those occurrences which no human agency could prevent. —*Med. Press & Circular.*

*The Artificial Leech.*—Mr. Stohlman, of the firm of Tieman & Co.. of New York, has invented what he terms the artificial leech. The apparatus consists : 1st, of a handle about six inches long, in which is concealed a knife that is operated by a spring the knife revolving with great rapidity, as soon as the " trigger" is touched, and making a painless incision about a quarter of an inch in diameter ; 2d. of any desired number of small glasses, with a capacity each of one or two fluid ounces ; by placing a few drops of ether in one of these glasses and plunging the glass into warm water, the contained air is rarefied and expanded ; the glass is now applied over the incision made by the knife and the air cooling down a vacuum is created and the blood in requisite quantity is easily drawn. The instrument is inexpensive, and will doubtless be useful, where leeches can not be obtained, or where they can not well be applied. Many have a repugnance to the leech that is incredible. In all such cases, this instrument will be useful and it is certainly very convenient.—*Richmond & Louisville Med. Journal*

---

*Three New Anæsthetics—Iodal, Bromal, and Bromiform.*—Scarcely has the name of chloral become familiar to the scientific world, when three new substances of the same character are announced by Dr. Rabuteau, of France (*Gaz. Hebdomadaire*, Oct. 22). Iodal is produced by treating iodine with alcohol and nitric acid. Bromal differs from chloral in having the chlorine of the latter replaced by bromine. Bromiform is made by decomposing bromal with potassa. In its chemical and anæsthetic qualities it is very analagous to chloroform and may be mistaken for it. But chloroform makes a violet colored solution of iodine, while that of bromiform is a magnificent carmine. Dr. Rabuteau is inclined to place the latter before chloroform. He thinks it will produce anæsthesia without a sleep so profound and dangerous. Bromal is irritating to the nose and eyes. Iodal has the same effect. The latter is unmanageable in consequence of boiling at 77° F. Injected in the rectum of a dog, it produced anæsthesia, followed by convulsions and death. The breath of the animal was strongly impregnated with the odor. The blood was found black, the flesh red, the spinal marrow and brain congested—presenting the same toxic effects as chloral.—*Pacific Med. & Surg. Journal.*

*Death from taking Chloroform into the Stomach.*—Dr. E. Fitzau (*Deutsche Klinik*, 1859, No 21) relates the case of a physician, 57 years of age, who swallowed 90 grms. of chloroform; he commenced immediately to stagger, like one intoxicated. After vomiting he sank into a deep stupor, attended with severe anæsthesia; his skin was pale and tolerably warm; his muscles were relaxed; his respiration short, and the action of his heart weak and intermittent. After the use of the stomach pump and different excitants, the epiglottis being depressed to prevent impending suffocation, an elastic tube was passed into the trachea, and immediately respiration became more free, and in about 14 hours sensibility returned. An acute gastritis became developed and rapid collapse ensued, and terminated in death at the end of 29½ hours from the time the chloroform was taken.—*Centralblatt f. d. Med. Wissenschaft.*—*Am. Jour. Med. Sciences.*

---

*Epileptoid Convulsions caused by Carious Teeth.*—By John W. Booth, M. D., of Tally-Ho, N. C.

October 7th, 1868, I visited an unmarried lady in an adjoining county in consultation with a respectable physician of that counf ty. She had epileptoid convulsious affecting principally some o- the muscles of the neck and the right arm. These attacks had been of almost daily occurrence for four years. She had nearly continuously during this time taken many and various remedies empirically, the cause of her affection not having been ascertained, without the slightest appreciable benefit. There was a very slight disposition to anæmia, and beyond this, there was not a symptom upon which to base a diagnosis or therapeutic indication. Appetite. digestion, general health all good, circulatory and generative apparatus acting normally.

In giving the history of her case, she stated that her first spell came on during an attack of toothache. Upon examining her mouth we found half a dozen carious teeth, and determined that to extract those teeth would afford the best chance of relief. Accordingly all the carious teeth were promptly extracted. There has been no return of the convulsions since. This lady has now improved considerably in flesh, and presents no anæmic phenomena. I need hardly add that her spirits are improved in an equal degree.—*American Jour. of Med. Science.*

*Substances Eliminated in the Breath.*—In answer to a "youthful physiologist" we desire to state that various substances besides carbonic acid are eliminated in the breath; such as chloride of sodium, nitrogen. hydrochlorate of ammonia, urate of soda, and urate of ammonia. In addition to these, the carbonate of ammonia is frequently, and carburetted hydrogen occasionally found in the breath; the former derived from decomposing matter in or between the teeth, the latter from the blood, into which it has entered from the alimentary canal. But besides the matters just mentioned, many odorous substances may exist in the breath, such as alcohol, the volatile principles of garlic, onions, and spices, camphor, ethers, chloroform, musk, and many other medicinal substances.

No specific name has been given to the organic matter found in the breath. It is known simply as "the organic substance in expired air." The substance in question is detected by passing expired air through strong sulphuric acid. It is albuminoid in constitution, has a fetid odor when accumulated in small and overcrowded rooms, and when allowed to putrefy, it becomes insufferably offensive. It has been suggested that this organic substance may be the medium or vehicle of certain contagions thrown off by the breath.

As to the circumstances under which the breath passes luminous from the nostrils, we reply that, when "phosporous is dissolved in oil and injected into the veins of an animal, it is given off by the lungs in some imperfectly oxidized state, so that the breath is luminous as it passes from the nostrils."—*Am. Eclectic Med. Review.*

---

*Irregular Dentition and Caries of the Lower Jaw.*—Dr. Pooley at the New York. Pathological Society, presented a small portion of the lower jaw removed by operation from a lady thirty years of age. The dentition of the patient had been very irregular; two of the second teeth of the lower jaw had never appeared, and the others that did were so uneven and deformed that they were extracted to make room for artificial ones, About a year ago she received a blow upon her chin, which was followed by considerable tenderness of the part, thought by her, however, to be principally due to pressure of the plate. Soon a couple of sinuses formed through which dead bone could be detected. An incision was made upon the part, with a view of removing the necrotic tissue. No loose bone was discovered, and the bare portion was removed by the saw and bone forceps. Two of the principal portions that were taken away contained, imbedded in their substance, a partially developed incisor, which as the result of the iritation caused by the injury, were the foci of the bone disease.—*Med. Record.*

---

*Toothache Amongst the Ancients.*—One by one our illusions as to the "good old times" vanish. Long had we cherished an idea that at least decayed teeth were unknown to our hardy ancestors, and were the peculiar privilege of our frivolous civilisation. Mr. Mummery, in an able paper before the Odontological Society, has shown, however, that teeth were at times unsound even when the ancient inhabitants of the British islands lived on coarse meal or the produce of the chase. Mr. Mummery has examined all the ancient skulls within his reach in order to determine this point. Beginning with the long-headed race, who are the earliest known human inhabitants, and have been supposed to be of a Basque type, be found few instances of real decay, not many of wearing down, and none of dental irregularity amongst sixty-eight Wiltshire skulls; whilst amongst the round-headed skulls from the same county, supposed to belong to the later Belgic immigrants whom Cæsar found in possession of the southern part of the island, there were many more cases of caries, more also of wearing away, and some of irregularity, which Mr. Mummery believes to be indicative of a coarse vegetable diet and scarcity of animal food. Oddly enough, in Yorkshire the skulls of the earlier or long-headed race exhibit many signs of dental disease, both caries, wear and tear, and signs of abscess. As for the Romans in Britain, the practice of burning their dead makes collecting of skulls by no means easy, yet out of 143 Britanno-Roman skulls 41 had carious teeth; irregularity and abscess were also common, but not wearing away. No traces of stopping or of artificial teeth have been found. Amongst Egyptian skulls wearing of the teeth is very common from the gritty, sandy character of the flour, and caries is by no means unfrequent. There are no traces of stopping, and it seems that the art of dentistry was almost confined to the extraction of teeth. Mr. Mummery's conclusion is that dental disease is not the exclusive privilege of a high state of civilisation.—*London Med. Times and Gazette.*

*Excessive Opium Eating.*—Dr. A. T. Schertzer, of Baltimore, Md., relates the case of a lady, twenty-eight years of age, who had consumed in two years, *five thousand eight hundred and forty ounces* of laudanum, and spent *eleven hundred and sixty dollars* in purchasing it. Without pretending to hold her attending physician to an unjust responsibility, the question naturally occurs, as to whether or not this heroic consumption and lavish expenditure might not have been prevented by the employment of judicious means, in the first instance? Medical men should always bear in mind that this dangerous habit is easily acquired and should guard as far as possible against it.

"While attached to the Medical Corps of the Navy," says Dr. S., "I have frequently met with natives of the East Indies who consumed opium with a voracity equal to that of the lady in question, but have never known a similar instance in this country."—*Med. and Surg. Reporter.*

